# Network analysis of adverse event patterns following immunization with mRNA COVID-19 vaccines: real-world data from the European pharmacovigilance database EudraVigilance

**DOI:** 10.3389/fmed.2025.1501921

**Published:** 2025-02-19

**Authors:** Renato Ferreira-da-Silva, Mariana Fernandes Lobo, Ana Margarida Pereira, Manuela Morato, Jorge Junqueira Polónia, Inês Ribeiro-Vaz

**Affiliations:** ^1^Porto Pharmacovigilance Centre, Faculty of Medicine of the University of Porto, Porto, Portugal; ^2^RISE-Health, Department of Community Medicine, Information and Health Decision Sciences (MEDCIDS), Faculty of Medicine of the University of Porto, Porto, Portugal; ^3^Allergy Unit, Instituto and Hospital CUF, Porto, Portugal; ^4^Laboratory of Pharmacology, Department of Drug Sciences, Faculty of Pharmacy of the University of Porto, Porto, Portugal; ^5^LAQV@REQUIMTE, Faculty of Pharmacy of the University of Porto, Porto, Portugal; ^6^RISE-Health, Department of Medicine, Faculty of Medicine of the University of Porto, Porto, Portugal

**Keywords:** COVID-19, mRNA vaccines, pharmacovigilance, adverse drug events, post-marketing drug surveillance, adverse drug reaction reporting systems

## Abstract

**Objective:**

To analyses real-world safety data of mRNA COVID-19 vaccines within the European Economic Area (EEA), using Individual Case Safety Reports (ICSR), and to evaluate the variability in safety profiles between different vaccine versions.

**Methods:**

We utilized EudraVigilance data from 1 January 2020, to 31 December 2023, focusing on Moderna (Spikevax) and Pfizer/BioNTech (Comirnaty) vaccines against COVID-19. We performed descriptive statistics, co-occurrence analysis, and correspondence analysis to identify patterns and clusters of adverse events following immunization (AEFI).

**Results:**

We retrieved 993,199 ICSR (Moderna: 394,484; Pfizer: 605,794), with most reports related to women patients (69%) and non-healthcare professionals (65%). A total of 10,804 distinct AEFI terms were described across the retrieved ICSR, with a cumulative occurrence frequency of 3,558,219 (Moderna: 1,555,638; Pfizer: 2,031,828). The most prominent serious clusters included headache, fatigue, pyrexia, myalgia, arthralgia, malaise, nausea, and chills, which frequently co-occurred with vaccination failure. Specific AEFI like fever, chills, malaise, arthralgia, injection site pain, inflammation, and warmth were more often linked to Moderna, while Pfizer was more commonly associated with vaccination failure, menstrual disorders (heavy menstrual bleeding and dysmenorrhea), and hypoesthesia. In older adults, serious clusters included confusional states, cerebrovascular accidents, and myocardial infarctions, while myocarditis and pericarditis were noted in younger males. Although rare, serious systemic AEFI, like anaphylactic reactions, were identified but require further causality evaluation.

**Conclusion:**

The overall safety of mRNA COVID-19 vaccines for mass vaccination is supported, but continuous pharmacovigilance remains essential. Identified clusters of AEFI, particularly serious and systemic ones, although rare and potentially influenced by other underlying causes, underscore the need for continuous monitoring and further epidemiological investigations to explore potential causal relationships.

## Highlights

• The most frequently reported adverse events following immunisation (AEFI) clusters associated with COVID-19 vaccines align with the common reactogenic profile of any vaccine, including local and systemic reactions such as headache, fatigue, pyrexia, myalgia, chills, malaise, nausea, arthralgia, dizziness, vaccination site pain, detected during clinical trials.
• Some clusters of serious systemic AEFI were identified, such as anaphylactic reactions, confusional states, cerebrovascular accidents, myocardial infarctions in older adults, and myocarditis in younger males; all are rare, and causality has not been evaluated.
• Although clusters of serious AEFI were identified, their rarity and potential underlying causes support the continued use of mRNA COVID-19 vaccines in mass vaccination programs due to a favorable benefit-risk ratio.
• Identifying AEFI clusters aids in understanding potential associations and underlying mechanisms, enhancing vaccine safety monitoring and predicting additional AEFI that might emerge over time.
• Insights from AEFI patterns in clinical trials, combined with post-marketing data, can inform patient stratification for each vaccine, including tailored safety assessments for specific subgroups such as the pediatric population, improving personalized recommendations and management strategies.

## Introduction

The pandemic caused by the Severe Acute Respiratory Syndrome Coronavirus 2 (SARS-CoV-2) has infected millions globally. This virus exhibits a more lethal nature compared to most other coronaviruses, except for those with a high severity profile like MERS, likely due to its effective human-to-human transmission and the initial absence of immunity within populations (1, 2). After the end of the pandemic emergency, there is a possibility that this virus could become endemic, making vaccination the key strategy for curtailing the spread and impact of SARS-CoV-2, as well as preventing long COVID-19 following infection (3–5). As of August 2024, *Our World in Data* reports that 70.7% of the global population has received at least one dose of a COVID-19 vaccine, with a total of 13.72 billion doses administered worldwide, of which 977.14 million were administered in the European Union (6).

The rapid spread of COVID-19 has necessitated the accelerated development and deployment of innovative vaccine technologies (7). Among the technological advancements, mRNA vaccines have been notably quick to produce and distribute (7, 8). In late 2020 and early 2021, the European Medicines Agency (EMA) granted emergency use authorizations for two mRNA vaccines: Pfizer/BioNTech’s (BNT162b2) in December and Moderna’s (mRNA-1273) in January (9). COVID-19 mRNA vaccines employ lipid nanoparticles to deliver a messenger RNA sequence into host cells, which then use this sequence as a template to produce the coronavirus spike protein, triggering an immune response without the introduction of the actual virus into the host system (10). Phase 3 randomized controlled trials (RCT) for the mRNA COVID-19 vaccines have demonstrated their robust safety profile (11, 12). Common adverse events following immunization (AEFI), included local reactions as pain and erythema, and systemic reactions, like fever, headache, and fatigue, all of which were more prevalent following the second dose. Despite undergoing clinical trials, these products face safety data limitations due to restrictive participant criteria and short study durations, which challenge the detection of rare, serious AEFI. Moreover, following their approval, the rapid deployment of mRNA vaccines has highlighted their potential and ongoing public safety concerns stemming from their novelty and swift production timeline (13).

Real-world application of COVID-19 vaccines has unveiled complexities not fully captured in initial clinical trials, which generally reported mild to moderate severity and quickly resolved adverse drug reactions (ADR). Vaccination efforts have expanded to a broader and more diverse range of populations - including the elderly, children, adolescents, pregnant and breastfeeding women, and the immunocompromised - and the importance of ongoing post-marketing safety surveillance has intensified (14–16). While post-marketing studies have largely affirmed the robust safety profile observed in early trials, emerging safety concerns such as anaphylaxis (17), Guillain-Barré syndrome (18), myocarditis and pericarditis (19), and thrombosis with thrombocytopenia syndrome (20) necessitate careful observation and management. In particular, the pediatric population requires specific attention in pharmacovigilance efforts, as adverse events such as myocarditis, although rare, have been reported, especially among adolescent males (21, 22). This underscores the necessity for tailored safety assessments and verification of adverse events post-vaccination in this vulnerable group. Moreover, the bioethical dimension of pediatric vaccination highlights the importance of balancing individual health protection with public health imperatives, while ensuring adherence to principles of informed consent and the protection of children’s rights as emphasized in recent discussions (23). Also, factors associated with unknown AEFI following mRNA COVID-19 vaccination, such as age, gender, and existing diseases or high-risk subgroups, have not been well established (24–26).

A recent study enhanced our understanding of mRNA COVID-19 vaccine safety by identifying five distinct AEFI clusters: infection, cardiac, respiratory/thrombotic, systemic, and nervous system (27). Infection-related AEFI might suggest vaccine inefficacy, while cardiac AE were more common among young males (21), consistent with prior research, and respiratory and systemic AE underscore the vaccines’ complex physiological impacts. Neurological disorders present another area of underexplored safety signals, encompassing a wide range of signs and symptoms from headaches to transverse myelitis, many of which are poorly characterized in existing studies (28–32). Although this study identified subgroups of AEFI, it did not explore the complex interactions between these events, focusing solely on the identification of isolated clusters.

Additionally, other emerging safety concerns have been noted in specific groups, such as menstrual disorders in women of reproductive age, including menorrhagia, metrorrhagia, and polymenorrhea (33, 34). Although some of these disorders are documented in the literature, there remains a significant gap in understanding how various AEFI co-occur within these clinical entities, as not all affected women experience just a single symptom. Despite the robust global deployment and surveillance of mRNA COVID-19 vaccines, the comprehensive analysis focusing on the concomitant occurrence of these AEFIs remains sparse. This lack of detailed exploration into how AEFIs cluster and interact in various population subsets, and the failure to map out adequately these events’ patterns, represents a critical gap in our current vaccine safety literature.

In this study, we aimed to delve into the real-world safety of mRNA COVID-19 vaccines within the European Economic Area (EEA), utilizing Individual Case Safety Reports (ICSR) to identify and characterize patterns and clusters of AEFI based on their frequency of co-occurrence. Secondarily, we evaluated the variability in safety profiles between different versions of vaccines.

## Materials and methods

### Pharmacovigilance data source

This real-world retrospective study utilized the EudraVigilance database, managed by the EMA, which serves as a comprehensive European data processing network collecting information on suspected AE from the use of marketed products within the EEA. The ICSR, sourced from regulatory and voluntary submissions, contains detailed information about the reported cases, including patient demographics, drugs involved, and AE. EudraVigilance is divided into two modules: the Post-Authorization Module (EVPM), which gathers reports of AE associated with EMA-authorized medicinal products, and the Clinical Trial Module (EVCTM), which collects data on AE that occur during clinical trials for products still under investigation. For our study, we exclusively used data from the EVPM. It is important to note that no causality assessment is made during report processing; therefore, all suspected ADR are better referred to as AE (in this case, specifically AEFI) and will be referred to as such throughout this manuscript.

### Study variables

Our study focused on ICSR of suspected AEFI associated with mRNA COVID-19 vaccines - specifically Moderna (mRNA-1273; Spikevax) and Pfizer/BioNTech (BNT162b2; Comirnaty; hereafter referred to as “Pfizer”). We retrieved ICSR data encompassing the period between the gateway receipt date (i.e., when the first ICSR was received) and December 31, 2023. No exclusion filters were applied during data extraction, ensuring a comprehensive dataset. The dataset encompassed both the original two formulations and all subsequent versions of each vaccine, totaling eight vaccine versions analyzed (Supplementary Table 1).

From each ICSR the following information was considered: a unique identifier number, the report type (spontaneous or non-spontaneous), the gateway receipt date, the primary source qualification (healthcare or non-healthcare professional), the primary source country (European or non-European Economic Area), the literature reference if available (some ICSR could indeed be already published as a case report), the patient age group, the patient sex, and a list of AEFI including their duration, outcome, and seriousness (35). The reported AEFI are coded as Preferred Term (PT) according to the Medical Dictionary for Regulatory Activities (MedDRA) (36), the international medical terminology developed under the auspices of the International Council for Harmonisation (ICH) of Technical Requirements for Pharmaceuticals for Human Use. Regarding the seriousness, a case is defined as serious by the ICH E2D guidelines (37) when it either results in death, is life-threatening, requires/prolongs hospitalization, results in persistent or significant disability/incapacity, is a congenital anomaly/birth defect, or results in some other clinically important condition.

The study follows the STROBE (Strengthening the Reporting of Observational Studies in Epidemiology) guidelines (Supplementary Table 2) (38). Additionally, the study adheres to the methodological standards outlined in the European Network of Centres for Pharmacoepidemiology and Pharmacovigilance (ENCePP) Guide on Methodological Standards in Pharmacoepidemiology (39).

### Statistical analysis

Descriptive statistics of ICSR were carried out globally, by year, and by vaccine version, based on the absolute and relative frequencies. The top 50 occurring AEFI terms were ascertained and visually represented using bar plots breakdown by seriousness.

Given the large number of distinct AEFI terms reported globally (*n* = 10,840), subsequent analyses were implemented considering only a subset of AEFI terms. Specifically, we consider AEFI that occurred in 1% or more of ICSR in at least one vaccine version, reducing the list of unique AEFI further analyzed to 120 terms.

### Co-occurrence analysis

Co-occurrence analysis was carried out to understand which pairs of AEFI co-occur more frequently than expected by chance. This type of analysis is commonly used in text analyses to identify words or phrases that frequently appear together or in biomedical research to identify medical terms that often appear together in scientific studies (40, 41). We used this type of analysis to abstract associations between different AEFI and, hence, potentially obtain insights about related side effects or underlying causes of adverse events. To this end, we computed a co-occurrence matrix. This is a squared symmetric matrix containing the frequency of co-occurrences of pairs of AEFI in the off-diagonal and, on the diagonal, the frequency of each AEFI.

For each pair of co-occurring AEFI, we defined a 2 × 2 contingency table describing their marginal and conditional distributions. The hypergeometric test was used to test statistically significant co-occurring pairs of AEFI (41, 42), where the null hypothesis assumes the independence of the number of ICSR with one AEFI and the number of ICRS with another AEFI, and the alternative hypothesis is that the co-occurrence of the two AEFI is overrepresented. The significance level was set at 0.05.

Statistically significant co-occurrences were visualized using network graphs created with the R package “visNetwork.” In these networks, edges between nodes representing pairs of AEFIs were pruned if the co-occurrence frequency was below the second quartile, or if the AEFIs occurred in fewer than 100 ICSR. Co-occurrence analysis was implemented on the overall sample and subsamples of ICRS based on the seriousness of AEFI and the age of vaccine recipients.

### Correspondence analysis

We additionally used correspondence analysis (CA) to understand if and which AEFI terms (or sets of terms) were more associated with some vaccine version than others. This technique is an extension of principal component analysis and is well suited to abstract relationships between categorical variables, such as type of vaccine version and AEFI, providing a means to visualize graphically the association between elements of the two variables (43). To this end, we built a contingency table in which the two groups of vaccines (Moderna and Pfizer) or the eight vaccine versions (versions 1–4 from Moderna and versions 5–8 from Pfizer, with correspondence details found in Supplementary Table 1) were listed as columns and all unique AEFI (PT terms) were listed as rows. We then filled in the table with counts of how often each AEFI term was reported with each vaccine and calculated the “average row profile,” which is determined by averaging row profiles with weights based on marginal row frequencies, and the “average column profile” by averaging column profiles, weighted by marginal column frequencies (44). Since ICSR may include several AEFI and several suspected vaccine versions, the same ICSR could be counted in multiple contingency table cells. Therefore, to ensure that association between vaccine versions and AEFI abstracted from the CA were not biased, for the CA only, we excluded ICSR with multiple suspected vaccine versions.

Correspondence analysis was performed on the contingency table, retaining the two dimensions with the highest explained inertia. Inertia can be viewed as a measure of variance among individual profiles relative the average profile. Each dimension is characterized by an eigenvalue, which indicates the amount of total inertia explained by that dimension, and corresponds to a principal axis or factor. The eight vaccine versions and the fifty AEFI that contribute the most in explaining the variance were represented in a biplot in which rows and columns are represented according to retained dimensions, and their relationship can be inferred from their relative spatial positions. Essentially, the further away from the center of the biplot and the closer an AEFI is to a vaccine version, the stronger their association. We looked for patterns or clusters of AEFI (rows) and vaccines (columns) that suggest commonalities or significant associations. This analysis informed us on the most relevant AEFI terms, differentiating the report patterns across different vaccine versions. Correspondence analysis was implemented using the function “CA” of the R package “FactoMineR,” and graphical displays of results were obtained with Excel and the R package “factoextra.”

## Results

### Characteristics of Individual Case Safety Reports (ICSR)

We retrieved 993,199 ICSR (Moderna: 394,484; Pfizer: 605,794) reported between 1 January 2020, and 31 December 2023, with at least one suspected COVID-19 vaccine. Of these, 8,679 (0.9%) were associated with multiple suspected vaccines or vaccine versions, and 7,703 (0.8%) were extracted from the scientific literature. Over 80% of the ICSR analyzed were reported during 2021 and 2022, with a drop of over 70% in the number of ICSR in 2023 relative to 2022. Most reports involved women patients (70%) and were submitted by non-healthcare professionals (65%), regardless of the type of vaccine. A total of 10,804 distinct AEFI terms were described in the retrieved ICSR, with a cumulative occurrence frequency of 3,558,219 (Moderna: 1,555,638; Pfizer: 2,031,828). Overall, the median number of AEFI terms per ICSR was 2 (P25 = 2, P75 = 5), but Moderna vaccines-associated ICSR tended to describe more suspected AEFI per report when compared to Pfizer ICSR. A quarter of ICSR described only a single AEFI, and 10,684 suspected AEFI terms were very or extremely rare, being described in less than 1% of ICSR. Finally, forty percent of ICSR were associated with one or more AEFI that represented a serious outcome, generally not consisting of the worse conditions within serious outcomes (hospitalization: 8.9%, life-threatening condition: 1.9%, death: 1.3%) (Table 1 and Supplementary Table 3).

**TABLE 1:** Reports characteristics by the suspected vaccine [10,840 distinct Preferred Term (PT) terms reported].

Characteristic	Vaccine		Overall, *N* = 993,1991
	**Moderna,** ***N* = 394,484^1^**	**Pfizer/BioNTech,** ***N* = 605,794^1^**	
**Year**			
2020	0 (0%)	2,437 (0.4%)	2,437 (0.2%)
2021	177,882 (45%)	249,900 (41%)	427,551 (43%)
2022	187,764 (48%)	255,758 (42%)	439,496 (44%)
2023	28,838 (7.3%)	97,699 (16%)	123,715 (12%)
**Gender**			
Female	270,948 (70%)	419,151 (71%)	685,224 (70%)
Male	117,565 (30%)	173,009 (29%)	288,471 (30%)
**Age**			
0–1 month	116 (< 0.1%)	160 (< 0.1%)	275 (< 0.1%)
2 months–2 years	203 (< 0.1%)	312 (< 0.1%)	513 (< 0.1%)
3–11 years	223 (< 0.1%)	1,830 (0.3%)	2,053 (0.2%)
12–17 years	2,666 (0.7%)	18,275 (3.2%)	20,913 (2.2%)
18–64 Years	306,287 (82%)	463,449 (82%)	764,884 (82%)
65–85 years	57,980 (16%)	75,224 (13%)	131,710 (14%)
More than 85 years	6,574 (1.8%)	9,383 (1.7%)	15,884 (1.7%)
**Primary source qualification**			
Healthcare professional	132,173 (34%)	221,075 (36%)	351,601 (35%)
Non-healthcare professional	262,311 (66%)	384,719 (64%)	641,598 (65%)
**Primary source country for regulatory purposes**			
European Economic Area	296,227 (75%)	490,970 (81%)	783,462 (79%)
Non-European Economic Area	98,257 (25%)	114,824 (19%)	209,737 (21%)
**# of PT terms per report**	3 (2, 5)	2 (1, 4)	2 (2, 5)
**# of PT terms per report**			
1	92,320 (23%)	154,488 (26%)	246,347 (25%)
2	75,589 (19%)	177,066 (29%)	251,036 (25%)
3	55,858 (14%)	79,368 (13%)	132,931 (13%)
4–5	76,059 (19%)	95,440 (16%)	170,240 (17%)
6–10	77,005 (20%)	79,882 (13%)	155,843 (16%)
>10	17,653 (4.5%)	19,550 (3.2%)	36,802 (3.7%)
**# of reports with ≥1 PT term associated with**			
Seriousness outcome	144,714 (37%)	256,050 (42%)	396,259 (40%)
Death	6,842 (1.7%)	5,713 (0.9%)	12,506 (1.3%)
Life-threatening condition	8,628 (2.2%)	10,177 (1.7%)	18,727 (1.9%)
Hospitalization	42,715 (11%)	46,634 (7.7%)	88,774 (8.9%)
Disabling condition	14,723 (3.7%)	21,998 (3.6%)	36,465 (3.7%)
Another serious condition	106,192 (27%)	207,980 (34%)	310,148 (31%)

PT, Preferred Term. Missing values were encountered in the following variables: gender (19,504; Moderna: 5,971; Pfizer: 13,634), age (56,967; Moderna: 20,435; Pfizer: 37,161).

^1^*n* (%); Median (IQR).

**FIGURE 1:**
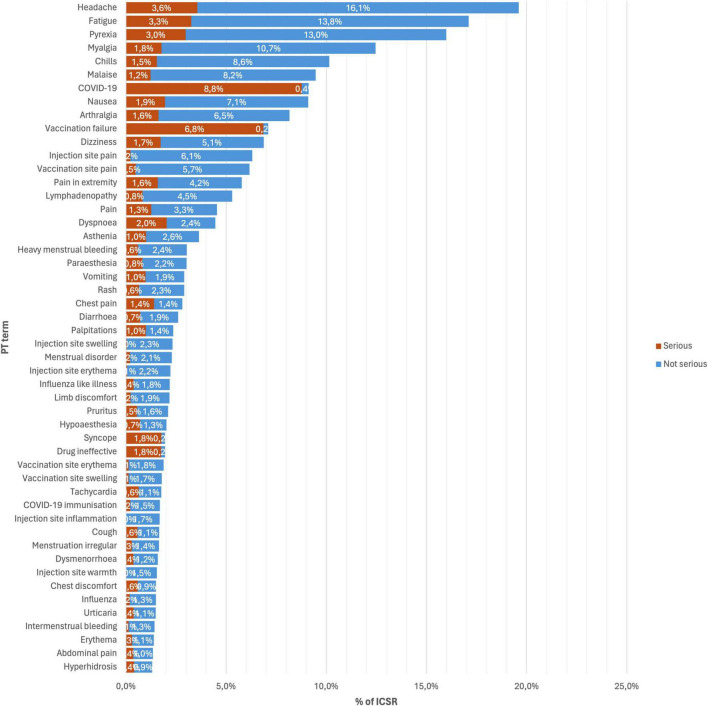
Top 50 occurring adverse events following immunization (AEFI) for all versions of mRNA COVID-19 vaccines.

**FIGURE 2:**
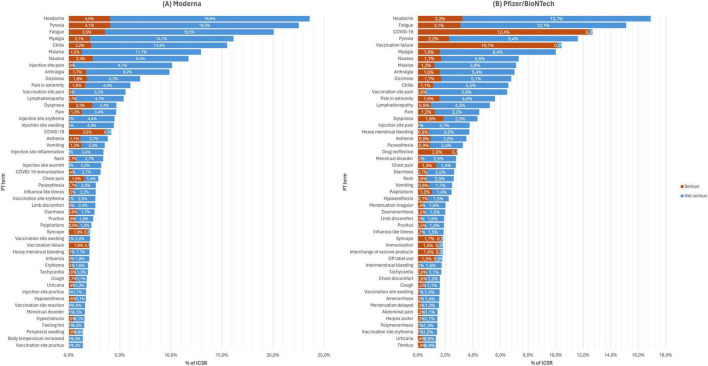
Top 50 occurring adverse events following immunization (AEFI) with panel **(A)** Moderna from 2021 to 2023, and **(B)** Pfizer/BioNTech from 2020 to 2023.

Figure 1 shows the top 50 occurring AEFI for all versions of mRNA COVID-19 vaccines, and Figure 2 shows the stratification by vaccine brand. Overall, systemic reactions such as headache, fatigue, and pyrexia were among the most frequently reported AEFI for both vaccines, with a higher propensity for serious outcomes in the Pfizer reports. In contrast, local reactions like injection site pain and erythema were more common but less frequently serious. For the Moderna (Figure 2A), the most frequently reported AEFI were headache (23.6%), pyrexia (22.5%), fatigue (20.1%), myalgia (16.1%), and chills (15.5%). Among these, headache, pyrexia, and fatigue had substantial proportions classified as serious, with 4.4, 3.5, and 3.5% of cases, respectively. Injection site reactions such as pain (10.1%), erythema (4.5%), and swelling (4.4%) were also commonly reported but predominantly non-serious. Similarly, nausea (11.7%) and malaise (13.0%) were frequently observed. For the Pfizer (Figure 2B), the most frequently reported AEFI were headache (16.9%), fatigue (15.1%), pyrexia (11.6%). COVID-19 was often co-reported with vaccination failure, likely reflecting cases where the vaccine was perceived as ineffective. Additionally, vaccination failure (10.4%) and the subsequent COVID-19 infection (12.7%) were frequently co-reported, with the latter likely being reported as a direct consequence of the former. Local reactions were also prevalent, with vaccination site pain (6.5%), pain in extremity (5.6%), and lymphadenopathy (4.3%) being (5.2%) frequently reported. Notably, COVID-19 and vaccination failure were reported in 10.2 and 8.4% of ICSR, respectively, with significant proportions considered serious. Other common systemic reactions included myalgia (10.0%), malaise (7.1%), and dizziness (6.8%).

### Co-occurrence analysis

**FIGURE 3:**
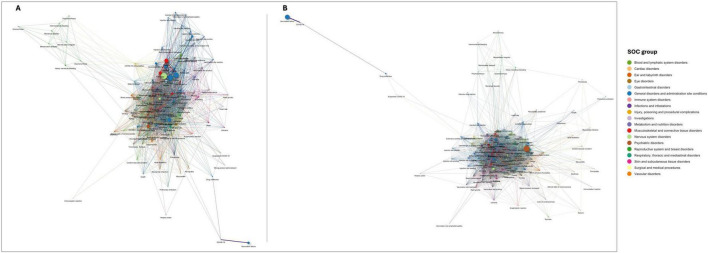
Networks of statistically significant co-occurrences: **(A)** overall AEFI terms; and **(B)** serious adverse events following immunization (AEFI) terms. Interactive versions are available in Supplementary Figures 1, 2, respectively.

Figure 3 exhibits the AEFI that co-occur together more frequently than expected by chance with Figure 3A depicting overall AEFI terms and Figure 3B the serious AEFI terms. Nodes represent AEFI and edges represent those AEFI that occur together. The size of nodes is proportional to the frequency of AEFI occurrence, the width of edges is proportional to the frequency of co-occurrence of pairs of AEFI terms, and the colors represent different SOC. This figure confirms that headache, fatigue, pyrexia, myalgia, arthralgia, malaise, nausea, and chills are the most frequently reported and commonly co-occurring AEFI globally. The next thicker edges include pain, injection/vaccination site pain, pain extremity, limb discomfort, asthenia, vomiting, dizziness, dyspnea, lymphadenopathy, and cough. Also, COVID-19 and vaccination failure seem to be highly correlated, co-occurring often and exclusively together. As depicted in the lower right corner of Figure 3A, the nodes corresponding to these AEFI are pulled away from the dense entangled main network of AEFI terms. Similarly, AEFI terms related to (1) menstrual disorders, (2) injection/vaccination site-related reactions, (3) rash and alike terms, and (4) cardiovascular/respiratory disorders, also seem to be pulled away from the center of the network, setting their own cluster of terms that tend to co-occur.

Figure 3B depicts the network of serious AEFI co-occurrences. Despite the edges being thinner relative to the overall network of AEFI (the sample of serious AEFI is smaller), this figure shows that among serious AEFI, the groups of AEFI composed by headache, fatigue, pyrexia, myalgia, arthralgia, malaise, nausea, and chills, and by COVID-19 and vaccination failure, remains the most frequently AEFI occurring together. However, in this figure, the cluster composed by off-label use, interchange of vaccine products and immunization presents edges as thicker as the previously mentioned clusters of AEFI. Confusional state stands out as the most frequently reported serious AEFI, significantly co-occurring with most of the reported serious AEFI. Moreover, death significantly co-occurs with myocardial infarction, cerebrovascular accident, confusional state, dyspnea, pneumonia, asthenia, and condition aggravated, potentially indicating the underlying causes of death. Anaphylactic reaction is significantly reported together with loss of consciousness, altered state of consciousness, blood pressure increase, heart rate increase, dyspnea, tachycardia, chest discomfort, feeling abnormal, abdominal pain, rash, rash erythematous, rash pruritic, erythema, pruritus, urticaria, specifying the type of manifestation of the anaphylactic reaction.

**FIGURE 4:**
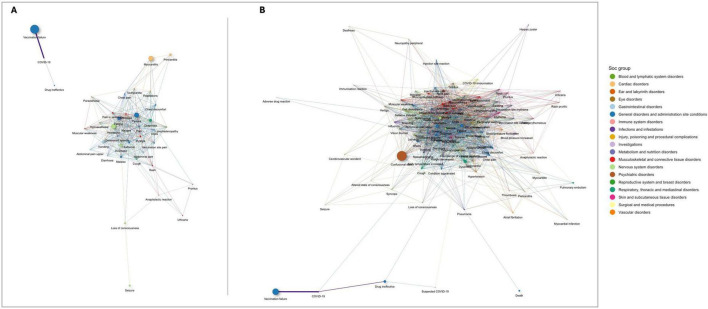
Networks of statistically significant co-occurrences of serious adverse events following immunization (AEFI): **(A)** ages 12–17 years; and **(B)** ages 64 and older. Interactive versions are available in Supplementary Figures 3, 4, respectively.

Figure 4 illustrates the network of statistically significant co-occurrences of serious AEFI for two age groups: (Figure 4A) ages 12–17 years, and (Figure 4B) ages 64 and older. Notable clusters in the group aged 12–17 years include vaccination failure and COVID-19, which are highly correlated and distinctly separated from other AEFI. Other significant clusters involve pain, injection site pain, systemic symptoms such as dizziness and dyspnea, and a prominent node for myocarditis, indicating frequent co-occurrence of these terms (Figure 4A). In the group aged 64 years and older (Figure 4B), the network is more densely connected, with confusional state, death, cerebrovascular accident, and myocardial infarction prominently co-occurring. COVID-19 and vaccination failure remain closely linked, similar to the younger age group. There is also a notable cluster of cardiovascular and respiratory disorders, including myocardial infarction and pulmonary embolism, which frequently co-occur with other severe conditions. Additionally, death is significantly associated with myocardial infarction, cerebrovascular accident, confusional state, and dyspnea (Figure 4B).

### Correspondence analysis

**FIGURE 5:**
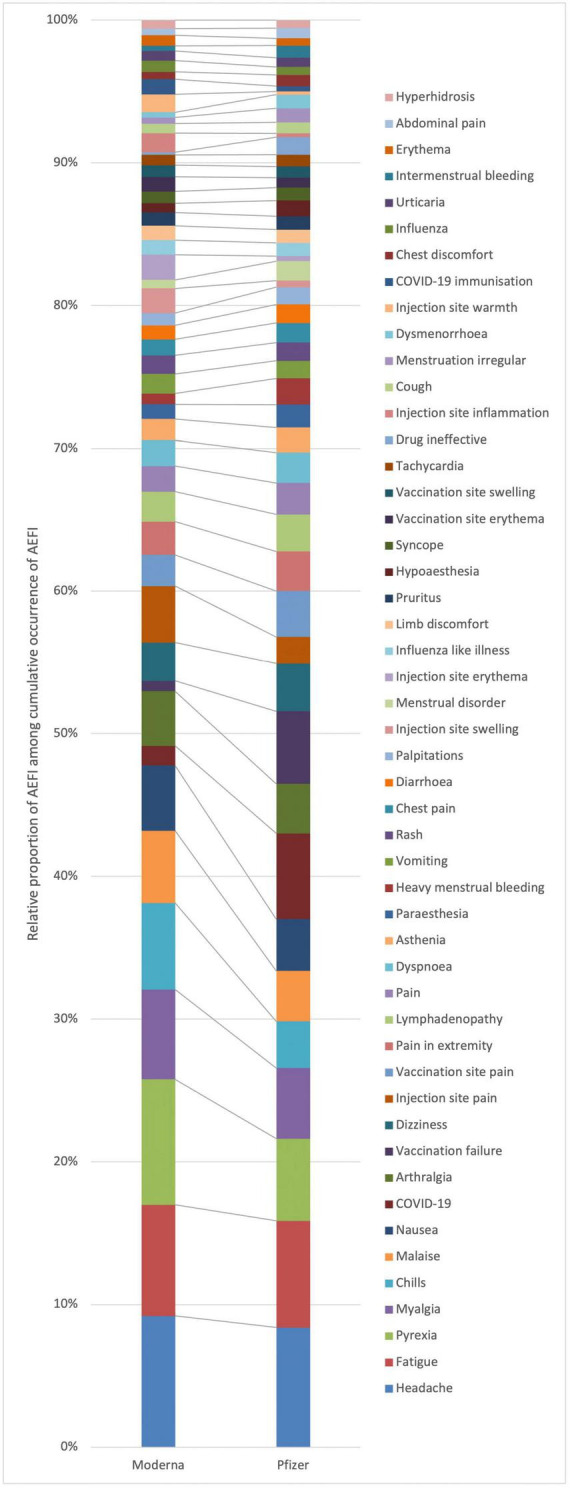
Row profiles of adverse events following immunization (AEFI) by vaccine group, displaying overall top 50.

Vaccine groups show slightly different patterns of the frequencies of reported AEFI (Figure 5). This means that compared to the overall row profile, some AEFI are more frequently reported with one vaccine group than the other. Particularly, Figure 5 shows that pyrexia, chills, malaise, arthralgia, injection site pain, injection site inflammation, and injection site warmth are more frequently associated with ICSR with Moderna. Conversely, COVID-19, vaccination failure, drug ineffective, vaccination site pain, heavy menstrual bleeding, menstrual disorders, dysmenorrhea, and hypoesthesia are more associated with Pfizer.

From the correspondence analysis, we retained dimension 1 and dimension 2, with eigenvalues 0.085 and 0.013, respectively. These two dimensions together account for 89% of the total variance (inertia) in the data. Moderna Version 1 (51%) and Pfizer Version 5 (44%) contribute most to the formation of dimension 1, whereas Pfizer version 6 contributes 83% to the formation of dimension 2. Therefore, these specific versions play a key role in defining dimensions 1 and 2, which capture the majority of the total variation (inertia) in our dataset.

**FIGURE 6:**
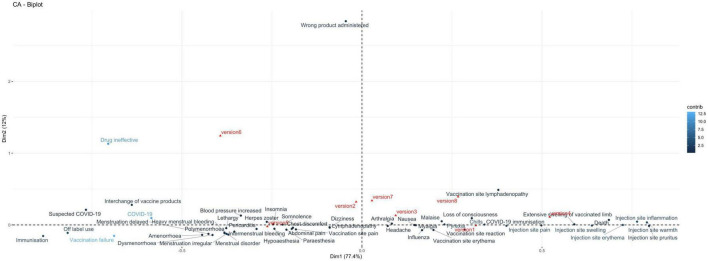
Correspondence analysis biplot illustrating the relationship between adverse events following immunization (AEFI) and vaccine versions. Vaccine versions are shown in red, including Moderna versions: version 1 (Original), version 2 (Omicron BA.1), version 3 (Bivalent Original/Omicron BA.4-5), and version 4 (Bivalent Original/Omicron XBB.1.5); and Pfizer versions: version 5 (Original), version 6 (Omicron BA.1), version 7 (Original/Omicron BA.4-5), and version 8 (Original/Omicron XBB.1.5). AEFI are shown in blue.

The biplot in Figure 6 shows that vaccine versions 5, 2, and 6 are separated from the rest on the left side regarding dimension 1; dimension 2 further separates vertically version 6 from other versions. Vaccination failure and COVID-19 are the AEFI that most significantly contribute to defining dimension 1 in our analysis. Positioned on the left side of this dimension and closely aligned with vaccine version 5, these AEFI indicate a strong association with this vaccine version, contributing to the overall variance explained by this axis. Moreover, immunization and off-label use are aligned with vaccine failure and aligned with version 5 suggesting an association between these AEFI and this vaccine version. On the other quadrant, vaccination site lymphadenopathy is aligned with versions 3 and 8. Injection site-related AEFI are clustered on the right side, thus potentially indicating a stronger association with versions 1, 4, and 3. Disorders related to menstruation are clustered on the left side of the biplot aligned with version 5, hence potentially more associated with this vaccine version. This is particularly more likely for AEFI that are further away from the biplot center, such as heavy menstrual bleeding, menstruation delayed, and amenorrhea. Also, arthralgia, malaise, nausea, headache, dizziness, and pyrexia are clustered toward the center of the biplot, indicating that there is not a predominantly association between these AEFI and any of the vaccine versions. Finally, the wrong product administered stands further apart from other AEFI, indicating no associations with other AEFI.

## Discussion

This study is the first to conduct a comprehensive post-implementation pharmacovigilance analysis using advanced co-occurrence and correspondence analyses on EudraVigilance data for mRNA COVID-19 vaccines, encompassing all vaccine versions and providing detailed evidence of AEFI patterns. Our findings reveal several safety concerns, notably the high frequency and co-occurrence of systemic reactions. Although regulatory authorities, healthcare professionals, and vaccine recipients already know most of these reactions, our study provides a detailed examination of their patterns. Indeed, our work offers new insights into how AEFI tend to co-occur, filling a gap in the scientific literature that previously relied solely on empirical clinical observation. Furthermore, safety evaluations in clinical trials were primarily limited to relatively healthy individuals, while real-world settings prioritized vaccinating vulnerable patients at high risk for severe COVID-19 (13, 45, 46). This study aimed to bridge the gap between clinical trials and real-world settings by identifying potential safety concerns that could become significant as vaccine coverage expands globally. Our comprehensive analysis enhances the understanding of AEFI associations and mechanisms, informing policy, regulatory, and clinical decisions, and refining risk stratification and vaccine protocols against COVID-19.

Our findings show that for all analyzed vaccines, most cases were reported between 2021 and 2022, primarily concerning women. The median number of AEFI per ICSR was two, with Moderna cases having more suspected AEFI than Pfizer. Over 40% of cases reported one or more serious AEFI. Systemic reactions like headache, fatigue, and fever were among the most frequently reported AEFI for both vaccines, with a higher propensity for serious outcomes in Pfizer reports. In contrast, local reactions like injection site pain and erythema were more common but less frequently serious. Some specific AEFI, such as fever, chills, malaise, arthralgia, injection site pain, inflammation, and warmth, were more frequently associated with Moderna’s vaccines. Conversely, AEFI such as vaccination failure/drug ineffectiveness, vaccination site pain, menstrual disorders (including heavy menstrual bleeding and dysmenorrhea), and hypoesthesia, were more commonly linked to Pfizer’s.

A meta-analysis of 12 clinical trials identified fatigue and headache as the most common AEFI, consistent with our findings (47). Other studies similarly reported headache, fatigue, and myalgia as frequent systemic events, with pain, erythema, and swelling at the injection site being common local events (48–51). It is also noted that reactogenicity and AEFI are more prevalent in participants receiving mRNA-based vaccines than other types (49). Additionally, Mathioudakis et al. (52) were the first to report a significant association between a prior COVID-19 infection and a significantly higher incidence and severity of self-reported non-serious side events following COVID-19 vaccination. Although we lack data on the prior infection status of patients in our study, it is likely that many cases involved previously infected individuals. Given the timing of the vaccination campaign, many of these individuals may have been asymptomatic or untested at the time of vaccination, leading to a temporal association between the vaccine and subsequent COVID-19 symptoms. This is critical, as pre-existing immunity from prior infection could amplify the immune response upon vaccination, leading to a higher frequency and severity of AEFI.

We also identified several clusters of AEFI that co-occur more frequently than expected by chance. These include (i) headache, fatigue, fever, myalgia, arthralgia, malaise, nausea, and chills, and (ii) vaccination site pain, limb pain/discomfort, asthenia, vomiting, dizziness, dyspnea, lymphadenopathy, and cough. The literature shows that for any authorized COVID-19 vaccine, whether mRNA or not, grade 1 severity for local and systemic events was most common, followed by grades 2 and 3 (53, 54). Injection site pain was the most frequent local AEFI, while fever, fatigue, and headache were the common systemic AEFI across mRNA vaccines (53, 55). Lee et al. (27) identified five distinct safety profiles - infection, cardiac, respiratory/thrombotic, systemic, and nervous system - that closely align with the AEFI clusters found in our study. In the infection AEFI cluster, the most reported issue was vaccine ineffectiveness, supporting our findings. For respiratory and thrombotic AEFI, symptoms such as cough and dyspnea appeared among our 50 most frequent PT terms. Although pulmonary embolism did not feature in these 50 most frequent terms, it emerged in our co-occurrence analysis, particularly among individuals aged 64 and older. In the cardiac AEFI cluster, myocarditis and pericarditis in young males were noted, while the systemic AEFI cluster, including headache and dizziness, was also consistent with our findings. Therefore, there is a chance that all vaccines induce some undesirable side effects that do not necessarily have a causal relationship specifically with mRNA vaccines. Typically, these events occur soon after the injection, are not life-threatening, and, except for extremely rare anaphylactic reactions, do not require additional treatments. The immune system’s activation and inflammatory processes that are expected as part of the body’s immune response to vaccination, similar to other vaccines, can lead to these common AEFI, reflecting a normal response to the antigenic stimulation provided by the vaccine (56, 57).

Mild sensory symptoms, such as numbness and tingling, are frequently reported in the literature, including cases of Bell’s palsy (58). While we did not find prominent clusters of these AEFI, other nociceptive experiences, such as vaccination site pain or systemic myalgias, were identified. These may redirect attention toward the body, potentially causing symptoms rather than indicating neurotoxic or immune-mediated processes. Other biological explanations, like small fiber neuropathy after the second dose of the Pfizer vaccine, have also been suggested in previous reports (59). Furthermore, pain at the vaccination site and limb discomfort are expected and well-documented reactions (60–66). These occur because the injection introduces vaccine components directly into muscle tissue, causing local inflammation and immune cell recruitment (67). Pain in the deltoid muscle is commonly experienced by COVID-19 vaccine recipients within a few hours after receiving the shot. This pain may be due to the leakage of protein-rich fluid and produced antigens at the site of tissue damage (68). While these reactions can cause discomfort, they are generally mild to moderate in severity and resolve on their own, posing minimal risk to the overall safety of the vaccines. This localized immune response is a standard mechanism following immunization, aimed at initiating a robust and effective systemic immune reaction.

Among other clusters of interest, on the periphery of the main network, menstrual disorders emerged as a significant cluster - a signal long identified in the literature, even if not all studies have proven causality (69–74). There is a heterogeneity in the menstrual abnormalities’ conditions such as menorrhagia, metrorrhagia, polymenorrhea, amenorrhea, and heavy menstrual bleeding. Our analysis of extreme age groups (12–17 and over 64 years) did not identify this cluster of AEFI, aligning with the understanding that these events are more commonly observed in the broader reproductive age range, typically beyond adolescence, as supported by other published studies (33). However, it has also been reported in some literature specifically within the 12–15 age group (75). Kadri et al. (34) estimated in a meta-analysis that the pooled prevalence of menorrhagia, polymenorrhea, abnormal cycle length, and oligomenorrhea to be 24.2, 16.2, 6.6, and 22.7%, respectively. Menstrual disorders, such as heavy menstrual bleeding, can stem from hormonal and physiological disruptions or anatomical abnormalities (76). The systemic immune response post-vaccination, which includes modulation of the immune environment, may interfere with hormonal and inflammatory pathways involved in the menstrual cycle, explaining the association between vaccination and menstrual changes (10). Previous research has shown that certain vaccines can affect the immune and hormonal environment, supporting this hypothesis (77–79). Additionally, immune cell activity varies across menstrual phases, potentially contributing to these issues (80). While generally mild and short-lived, women and healthcare professionals need to be aware of these potential reactions (76), which appears to have emerged specifically with this vaccine, as there is no evidence in the literature of similar AEFI linked to other vaccines.

Our study also identified clusters of serious AEFI that typically co-occur, with the most frequent being headache, fatigue, fever, myalgia, arthralgia, malaise, nausea, and chills. Systemic-type reactions are predominant, even though some local reactions are included within these clusters. In fact, systemic reactions to COVID-19 vaccines, such as fatigue and headache, are frequently documented in cohort event monitoring, clinical trials, and spontaneous reports among adults, children, and adolescents (12, 81–87). Kim et al. (88) reported that headache, pyrexia, fatigue, nausea, chills, and myalgia were among the most common AEFI in recipients of the Pfizer vaccine, mirroring our observations. Although the severity of these AEFI varies across studies, with some classifying them as non-serious, their high frequency and co-occurrence strongly indicate a consistent pattern of systemic response to the vaccine. Understanding the molecular mechanisms behind these AEFI is essential for exploring ways to reduce reactogenicity while preserving vaccine efficacy.

Our data also underscore the importance of monitoring less common but serious AEFI, such as anaphylaxis, which, although rare, is potentially life-threatening and requires immediate medical attention. In fact, similar to other medications, vaccines can sometimes cause hypersensitivity reactions, which are typically rare and mostly mild (89, 90). Vaccine-induced anaphylaxis is estimated to occur at a rate of approximately 1 per million doses and it can be triggered by the active substance or by other components (e.g., egg, gelatin) (91). When an allergic reaction to a vaccine occurs, it can be challenging to determine whether the vaccine itself or its excipients or inactive ingredients are responsible (92). With regards to mRNA vaccines, polyethylene glycol (PEG) and tromethamine have emerged as potential suspected anaphylaxis triggers (93, 94). However, most studies consistently reported a lack of reaction reproducibility (with tolerance to subsequent doses) and negative allergy tests, pointing away from an IgE-mediated allergy (95). In fact, a meta-analysis of 1,366 individuals with an immediate reaction to the first COVID-19 mRNA vaccination found that only six patients had severe immediate reactions in the second vaccination, and 232 had mild symptoms (96). Several mechanisms apart from IgE-mediated allergy were described as potential contributors to mRNA vaccine-induced reactions, including non-IgE mediated reactions to PEG (97), contact system activation by nucleic acid or direct mast cell activation (98). Nevertheless, the precise mechanism is still unknown. Moreover, in spite of initial reports of a higher than expected incidence of anaphylaxis (reaching 11.1 per million doses administered for Pfizer/BioNTech vaccine in December 2020) (99), it became progressively clear that the global incidence is low and comparable to the incidence of anaphylaxis associated with other traditional vaccines, reinforcing the relative safety of COVID-19 vaccines (90, 100, 101). The lack of consistency in the definition of anaphylaxis and the initial use of more sensitive definitions (classifying as anaphylaxis reactions that would not be considered as so using the usual clinical criteria), might have contributed to the initial figures (102). In our study, within the cluster of anaphylactic reactions, AEFI such as loss of consciousness, increased blood pressure and heart rate, rash, erythema, pruritus, and urticaria are commonly associated. These symptoms are consistent with the clinical presentation of anaphylaxis and are likely not additional AEFI but part of the primary anaphylactic reaction (103, 104).

Moreover, our study identified notable age-related differences in serious AEFI. Myocarditis was predominantly observed in younger individuals, particularly males, which aligns with reports from other real-world data studies and clinical trials (105–108). Karlstad et al. (106) found the number of myocarditis and pericarditis in young-vaccinated males was 11.5 and 11.7 events per million doses administered for Pfizer and Moderna, respectively. Although the exact reason is unknown, researchers suggest that testosterone’s pro-inflammatory effects might make young males more prone to post-mRNA vaccine myocarditis, while estrogen is anti-inflammatory (109). Interestingly, two studies found an increased myocarditis risk from Pfizer in females (12–39 years), though the reasons are unclear. However, the myocarditis risk from Moderna remained higher in males than females (110, 111).

In older adults, serious AEFI, such as confusional states, death, cerebrovascular accidents, and myocardial infarctions, were identified as a cluster of co-occurrences. The confusional state stands out as the most frequently reported serious AEFI, significantly co-occurring with most of the reported serious AEFI in our data. It was particularly prevalent in the subgroup aged 64 and older, often co-occurring with death, cerebrovascular accident, and myocardial infarction. This pattern suggests potential underlying causes linking these severe outcomes in this age group. Furthermore, older individuals face an increased risk of cardiovascular and cerebrovascular disorders, including myocardial infarction and pulmonary embolism, due to pathogenic alterations in the vasculature, including atherosclerotic disease, hemorrhages, aneurysms, vascular cognitive impairment, and microcirculation disruptions (112–114). Various types of thrombosis, including deep vein thrombosis, pulmonary embolism, cerebral venous sinus thrombosis (CVST), and arterial or abdominal clots, have been reported in individuals 4–30 days after receiving mRNA vaccines, though at a lower frequency (115–117). A previous study using EudraVigilance data identified 26 cases of CVST with mRNA COVID-19 vaccines, none of which were associated with thrombocytopenia. Despite this, CVST was more frequently observed with the ChAdOx1 nCov-19 vaccine (117). Therefore, age-related changes in the inflammatory response, vascular function, and hemostasis may predispose older individuals to an exaggerated inflammatory response, thrombus formation, and endotheliopathy following COVID-19 vaccination (118). These vaccine-induced pathophysiological mechanisms could ultimately lead to a higher frequency of severe outcomes, such as lethal reactions, hospitalizations, and life-threatening events in this age group (113).

The strong co-occurrence between vaccination failure and COVID-19 in our study raises important considerations regarding potential issues with vaccine efficacy or reporting accuracy. This pattern was evident in both younger and older age groups COVID-19 was frequently reported as an AEFI, likely reflecting cases where the vaccine failed to prevent infection, resulting in the disease as a consequence of vaccination failure. Similarly to the cases of death, COVID-19 was likely reported as an AEFI when it is actually the outcome of another event. This likely occurred due to the challenges in gathering detailed information during the mass vaccination campaign, leading reporters to document the outcome (COVID-19 infection) rather than the primary cause (vaccination failure). Although our study does not focus on efficacy issues, previous or concurrent infection can be misinterpreted as vaccine failure, underestimating the true efficacy of the vaccine and highlighting the need for a more careful analysis of reporting data. When healthcare professionals (or vaccine recipients) observe COVID-19 symptoms in a vaccinated patient, they may quickly attribute these symptoms to vaccine failure without considering the possibility of an undetected prior infection or an infection acquired shortly before or after vaccination. However, caution should be exercised because the primary goal of mRNA-based vaccines is to prevent severe disease and death, as demonstrated in several studies, not to prevent SARS-CoV-2 infection (11, 12, 119, 120). Vaccination against COVID-19 will not reduce deaths from other causes, such as unrelated health problems. Therefore, during vaccination campaigns, it is expected that deaths from other causes will continue to occur, sometimes closely following vaccination, without necessarily being related to it. Despite the emergence of variants like Omicron, which have reduced vaccine effectiveness against symptomatic infections, these vaccines still provide robust protection against severe outcomes (121, 122). The vaccines maintain a high effectiveness against severe disease and hospitalization, with rates ranging from 80 to 100% following a booster dose and remaining stable over time (123). Future research should explore the temporal relationship between these AEFI to rule out the possibility that patients were already infected, albeit asymptomatically, at the time of vaccination. Furthermore, it is important to acknowledge that the use of mRNA vaccines occurred across diverse epidemiological contexts, marked by the circulation of different SARS-CoV-2 variants over time. Variants such as Alpha, Delta, and Omicron introduced changes in viral transmissibility, immune evasion, and disease severity, potentially influencing both vaccine effectiveness and safety profiles (124). However, in our study, it was not possible to stratify adverse event patterns according to the specific variant in circulation at the time of vaccination due to data limitations. This remains an important factor to consider in future research, as variant-specific dynamics may provide further insights into vaccine safety outcomes.

Additionally, evidence suggests that mRNA vaccines may reduce the risk of developing long COVID when administered prior to SARS-CoV-2 infection. A systematic review analysing data from over 17 million individuals found that vaccination, particularly with two doses, was associated with a lower likelihood of long COVID symptoms (125). While the mechanisms behind this effect remain unclear, hypotheses include reduced viral persistence and modulation of inflammatory and immune responses. However, most studies focused on short-term impacts, with limited data on booster doses. Future research should explore whether this protective effect differs between individuals with or without prior SARS-CoV-2 infection before vaccination. In our study, this relationship could not be assessed due to the lack of patient-specific data on infection history, particularly in relation to its potential impact on safety outcomes.

### Study weaknesses and strengths

Taking into account the inherent limitations of spontaneous reporting systems such as EudraVigilance, the findings of our study must be interpreted with caution. Firstly, causality between the reported AEFI and the vaccines was not established. The reported events are based on suspicion and may be influenced by other factors such as underlying diseases or interactions with other medications. Secondly, spontaneous reporting systems like EudraVigilance are subject to underreporting, a well-known limitation that can introduce reporting biases, thus limiting the ability to estimate the true incidence of AEFI. Additionally, the inherent subjectivity of the reporting process can lead to over-representation or misclassification of certain patterns. For instance, some adverse events may represent overlapping signs and symptoms of a single clinical entity rather than distinct events (e.g., symptoms such as dyspnea, rash, and hypotension being reported separately, despite all being part of an anaphylactic reaction). Thirdly, we did not directly compare safety profiles among different vaccines, as they were administered to distinct population subgroups, under varying epidemiological contexts and time frames. This variability adds complexity to the interpretation of observed patterns. Fourthly, our analysis did not differentiate between AEFI from initial doses versus booster doses due to the lack of systematically reported data on vaccination frequency in EudraVigilance. While such information may occasionally be present in individual case narratives, it was not accessible in the dataset we used. This limitation prevents the determination of dose-specific AEFI patterns and highlights the need for future studies with access to more granular data to address this gap. Fifthly, the lack of patient-specific data, such as prior infection status, constrains the interpretation of certain findings (e.g., co-occurrence of COVID-19 and vaccination failure). Without these data, it becomes challenging to disentangle whether certain events are related to vaccination, infection, or a combination of both. Lastly, the subjectivity in severity classification represents an additional limitation. Severity is often determined based on the reporter’s perception, rather than standardized clinical criteria (e.g., WHO severity guidelines). This subjectivity can lead to inconsistencies in reported severity patterns and may impact the observed distribution of serious versus non-serious events. These limitations are not unique to our study but rather systemic challenges of spontaneous pharmacovigilance data. Addressing these issues requires complementary approaches, including well-designed pharmacoepidemiological studies, to further validate and refine safety signals emerging from these datasets.

Despite these limitations, our study also has notable strengths. Firstly, it provides a comprehensive post-implementation analysis of mRNA COVID-19 vaccines’ safety using real-world data from a large and diverse population across the EEA. The large sample size not only enhances the generalizability of our findings but also helps to mitigate potential errors, providing a robust basis for safety assessments. Secondly, the use of advanced co-occurrence and correspondence analyses offers new insights into patterns of AEFI that were not evident from clinical trials alone or from conventional descriptive statistical analyses, which are typically limited to determining frequencies. Thirdly, identifying AEFI clusters aids in understanding potential associations and underlying mechanisms. This knowledge enhances vaccine safety monitoring and allows for predicting additional AEFI that might emerge over time. For example, based on AEFI observed during clinical trials, we can anticipate other related AEFI that patients might experience beyond the trial’s monitoring period. Additionally, understanding these AEFI patterns can inform the stratification of patients for each vaccine, potentially improving personalized vaccine recommendations and management strategies. Lastly, our findings support signal prioritization and triaging, guiding further investigations and public health responses, thereby ensuring the continued safety and efficacy of vaccination programs.

## Conclusion

Our findings enhance the understanding of mRNA COVID-19 vaccine safety profiles, supporting their acceptability for mass vaccination. The most frequently reported AEFI align with the common reactogenic profile of any vaccine detected during clinical trials. Identified clusters of serious systemic AEFI, although rare and potentially influenced by other underlying causes, underscore the need for continuous monitoring and further epidemiological investigations to explore potential causal relationships. This ongoing pharmacovigilance will help to predict additional AEFI that might emerge over time, to inform the stratification of patients for each vaccine, and to improve personalized recommendations and management strategies. Overall, these findings support the continued use of mRNA COVID-19 vaccines in mass vaccination programs based on a favorable benefit-risk ratio.

## Data Availability

The raw data supporting the conclusions of this article will be made available by the authors, upon reasonable request.
