# Supplementary material for: Network analysis of adverse event patterns following immunization with mRNA COVID-19 vaccines: real-world data from the European pharmacovigilance database EudraVigilance
Source: Front Med (Lausanne). 2025 Feb 19;12:1501921. doi: 10.3389/fmed.2025.1501921 (PMC11879978; doi:10.3389/fmed.2025.1501921)
Supplement: Supplementary file 2 [file Table_1.pdf]

# Supplementary Material

**Table S1.** Overview of mRNA COVID-19 vaccine formulations and their target variants.

| Vaccine Brand | Version | Variant Description | Formulation Detail[1] | Gateway Receipt Date[2] |
|---|---|---|---|---|
| **Moderna (mRNA-1273; Spikevax)** | 1 | Original | Contains elasomeran, a molecule called messenger RNA (mRNA) with instructions for producing a protein from the original strain of SARS-CoV-2. | 2021 |
| | 2 | Bivalent Original/Omicron BA.1 | Contains elasomeran and an additional mRNA molecule, imelasomeran, with instructions for producing a protein from the Omicron BA.1 subvariant of SARS-CoV-2 | 2022 |
| | 3 | Bivalent Original/Omicron BA.4-5 | Contains elasomeran and an additional mRNA molecule, davesomeran, with instructions for producing a protein from the Omicron BA.4 and BA.5 subvariants of SARS-CoV-2. | 2022 |
| | 4 | Omicron XBB.1.5 | Contains andusomeran, an mRNA molecule with instructions for producing a protein from the Omicron XBB.1.5 subvariant of SARS-CoV-2. | 2023 |
| **Pfizer/BioNTech (BNT162b2; Comirnaty)** | 5 | Original | Contains tozinameran, a messenger RNA (mRNA) molecule with instructions for producing a protein from the original strain of SARS-CoV-2 | 2020 |
| | 6 | Original/Omicron BA.1 | Contains tozinameran and riltozinameran, an mRNA molecule with instructions for producing a protein from the Omicron BA.1 subvariant of SARS-CoV-2 | 2022 |
| | 7 | Original/Omicron BA.4-5 | Contains tozinameran and famtozinameran, an mRNA molecule with instructions for producing a protein from the Omicron BA.4 and BA.5 subvariants of SARS-CoV-2 | 2022 |
| | 8 | Omicron XBB.1.5 | Contains raxtozinameran, an mRNA molecule with instructions for producing a protein from the Omicron XBB.1.5 subvariant of SARS-CoV-2 | 2023 |

[1] The mRNA in these vaccines serves as a template for cells to produce a protein that mimics the SARS-CoV-2 virus protein, initiating an immune response without introducing the virus itself. This technology allows for rapid adaptation to different virus variants.

[2] Gateway Receipt Date refers to the date the first Individual Case Safety Report (ICSR) for each vaccine variant was received in the EudraVigilance database. No filters were applied to the data retrieval process.
