# Supplementary material for: Network analysis of adverse event patterns following immunization with mRNA COVID-19 vaccines: real-world data from the European pharmacovigilance database EudraVigilance
Source: Front Med (Lausanne). 2025 Feb 19;12:1501921. doi: 10.3389/fmed.2025.1501921 (PMC11879978; doi:10.3389/fmed.2025.1501921)
Supplement: Supplementary file 4 [file Table_3.pdf]

Supplementary Material

Table S3. Reports characteristics by suspected vaccine version (10840 distinct PT terms reported).

Characteristic	Vaccine								Overall, N = 993,199[1]
	Moderna, N = 394,484[1]				Pfizer/BioNTech, N = 605,794[1]				
	Original, N = 385,244[1]	Omicron XBB.1.5, N = 481[1]	Bivalent Original/Omicron BA.1, N = 8,102[1]	Bivalent Original/Omicron BA.4-5, N = 787[1]	Original, N = 585,566[1]	Omicron XBB.1.5, N = 4,386[1]	Original/Omicron BA.1, N = 6,660[1]	Original/Omicron BA.4-5, N = 11,045[1]	
**Year**									
2020	0 (0%)	0 (0%)	0 (0%)	0 (0%)	2,437 (0.4%)	0 (0%)	0 (0%)	0 (0%)	2,437 (0.2%)
2021	177,882 (46%)	0 (0%)	0 (0%)	0 (0%)	249,900 (43%)	0 (0%)	0 (0%)	0 (0%)	427,551 (43%)
2022	183,317 (48%)	0 (0%)	4,367 (54%)	112 (14%)	249,900 (43%)	0 (0%)	2,863 (43%)	3,243 (29%)	439,496 (44%)
2023	24,045 (6.2%)	481 (100%)	3,735 (46%)	675 (86%)	83,329 (14%)	4,386 (100%)	3,797 (57%)	7,802 (71%)	123,715 (12%)
**Gender**									
Female	264,984 (70%)	305 (69%)	5,323 (68%)	412 (56%)	405,904 (71%)	2,806 (66%)	4,727 (73%)	6,882 (64%)	685,224 (70%)
Male	114,656 (30%)	140 (31%)	2,494 (32%)	327 (44%)	166,479 (29%)	1,471 (34%)	1,743 (27%)	3,948 (36%)	288,471 (30%)
**Age**									
0-1 Month	116 (<0.1%)	0 (0%)	0 (0%)	0 (0%)	159 (<0.1%)	0 (0%)	0 (0%)	1 (<0.1%)	275 (<0.1%)
2 Months - 2 Years	195 (<0.1%)	1 (0.3%)	5 (<0.1%)	2 (0.3%)	304 (<0.1%)	3 (<0.1%)	2 (<0.1%)	3 (<0.1%)	513 (<0.1%)
3-11 Years	196 (<0.1%)	1 (0.3%)	1 (<0.1%)	25 (4.1%)	1,799 (0.3%)	10 (0.2%)	1 (<0.1%)	21 (0.2%)	2,053 (0.2%)
12-17 Years	2,628 (0.7%)	2 (0.5%)	1 (<0.1%)	35 (5.7%)	18,141 (3.3%)	14 (0.3%)	47 (0.9%)	81 (0.8%)	20,913 (2.2%)
18-64 Years	302,846 (82%)	181 (45%)	3,047 (56%)	270 (44%)	453,123 (82%)	1,844 (45%)	3,524 (70%)	5,766 (56%)	764,884 (82%)
65-85 Years	55,387 (15%)	191 (48%)	2,210 (41%)	254 (41%)	68,743 (12%)	1,967 (48%)	1,351 (27%)	3,944 (38%)	131,710 (14%)
More than 85 Years	6,360 (1.7%)	24 (6.0%)	166 (3.1%)	29 (4.7%)	8,575 (1.6%)	222 (5.5%)	134 (2.6%)	508 (4.9%)	15,884 (1.7%)

**Primary Source Qualification**									
Healthcare Professional	130,493 (34%)	162 (34%)	1,044 (13%)	517 (66%)	214,488 (37%)	1,406 (32%)	1,082 (16%)	4,544 (41%)	351,601 (35%)
Non-Healthcare Professional	254,751 (66%)	319 (66%)	7,058 (87%)	270 (34%)	371,078 (63%)	2,980 (68%)	5,578 (84%)	6,501 (59%)	641,598 (65%)
**Primary Source Country for Regulatory Purposes**									
European Economic Area	292,083 (76%)	18 (3.7%)	4,034 (50%)	95 (12%)	479,437 (82%)	2,571 (59%)	3,869 (58%)	5,329 (48%)	783,462 (79%)
Non-European Economic Area	93,161 (24%)	463 (96%)	4,068 (50%)	692 (88%)	106,129 (18%)	1,815 (41%)	2,791 (42%)	5,716 (52%)	209,737 (21%)
**# of PT terms per report**	3 (2, 5)	2 (1, 5)	3 (1, 6)	2 (1, 3)	2 (1, 4)	2 (2, 4)	4 (2, 6)	2 (2, 3)	2 (2, 5)
**# of PT terms per report**									
1	89,384 (23%)	177 (37%)	2,537 (31%)	247 (31%)	149,928 (26%)	1,080 (25%)	1,353 (20%)	2,156 (20%)	246,347 (25%)
2	74,344 (19%)	71 (15%)	1,027 (13%)	156 (20%)	171,267 (29%)	1,302 (30%)	1,160 (17%)	4,680 (42%)	251,036 (25%)
3	54,857 (14%)	59 (12%)	808 (10.0%)	192 (24%)	76,784 (13%)	514 (12%)	771 (12%)	1,655 (15%)	132,931 (13%)
4-5	74,368 (19%)	86 (18%)	1,543 (19%)	81 (10%)	92,200 (16%)	680 (16%)	1,343 (20%)	1,297 (12%)	170,240 (17%)
6-10	74,979 (19%)	78 (16%)	1,872 (23%)	88 (11%)	76,524 (13%)	687 (16%)	1,686 (25%)	1,024 (9.3%)	155,843 (16%)
>10	17,312 (4.5%)	10 (2.1%)	315 (3.9%)	23 (2.9%)	18,863 (3.2%)	123 (2.8%)	347 (5.2%)	233 (2.1%)	36,802 (3.7%)
**# of reports with ≥1 PT term associated with**									
Seriousness outcome	139,397 (36%)	465 (97%)	4,274 (53%)	706 (90%)	244,822 (42%)	2,353 (54%)	3,412 (51%)	7,253 (66%)	396,259 (40%)
Death	6,688 (1.7%)	15 (3.1%)	89 (1.1%)	52 (6.6%)	5,284 (0.9%)	97 (2.2%)	83 (1.2%)	258 (2.3%)	12,506 (1.3%)
Life-threatening condition	8,382 (2.2%)	27 (5.6%)	203 (2.5%)	24 (3.0%)	9,557 (1.6%)	130 (3.0%)	183 (2.7%)	322 (2.9%)	18,727 (1.9%)
Hospitalization	41,890 (11%)	71 (15%)	581 (7.2%)	187 (24%)	44,667 (7.6%)	331 (7.5%)	511 (7.7%)	1,195 (11%)	88,774 (8.9%)
Disabling condition	14,176 (3.7%)	37 (7.7%)	502 (6.2%)	20 (2.5%)	20,944 (3.6%)	190 (4.3%)	542 (8.1%)	349 (3.2%)	36,465 (3.7%)
Another serious condition	101,439 (26%)	424 (88%)	3,817 (47%)	635 (81%)	198,842 (34%)	1,921 (44%)	2,745 (41%)	6,232 (56%)	310,148 (31%)

PT: Preferred Term; [1] n (%); Median (IQR); **Note:** Missing values were encountered in the following variables: gender (19,504; Original (Moderna): 5,604; Omicron XBB.1.5: 36; Bivalent Original/Omicron BA.1: 36; Bivalent Original/Omicron BA.4-5: 285; Original (Pfizer/BioNTech): 13,183; Original/Omicron BA.1: 109; Original/Omicron BA.4-5: 190; Omicron XBB.1.5: 215); age (56,967; Original (Moderna): 17,516; Omicron XBB.1.5: 172; Bivalent Original/Omicron BA.1: 81; Bivalent Original/Omicron BA.4-5: 2,672; Original (Pfizer/BioNTech): 34,722; Original/Omicron BA.1: 326; Original/Omicron BA.4-5: 1601; Omicron XBB.1.5: 721.
